# Advances in scarless foetal wound healing and prospects for scar reduction in adults

**DOI:** 10.1111/cpr.12916

**Published:** 2020-10-15

**Authors:** Jia‐Li Yin, Yan Wu, Zheng‐Wei Yuan, Xing‐Hua Gao, Hong‐duo Chen

**Affiliations:** ^1^ Key Laboratory of Immunodermatology Ministry of Education Department of Dermatology The First Hospital of China Medical University Shenyang Liaoning China; ^2^ National and Local Joint Engineering Research Center of Immunodermatological Theranostics The First Hospital of China Medical University Shenyang Liaoning China; ^3^ Key Laboratory of Health Ministry for Congenital Malformation Shengjing Hospital China Medical University Shenyang Liaoning China

**Keywords:** fibroblasts, foetal wound healing, hypertrophic scars, non‐coding RNA, scarless model

## Abstract

Healing after mammalian skin injury involves the interaction between numerous cellular constituents and regulatory factors, which together form three overlapping phases: an inflammatory response, a proliferation phase and a remodelling phase. Any slight variation in these three stages can substantially alter the healing process and resultant production of scars. Of particular significance are the mechanisms responsible for the scar‐free phenomenon observed in the foetus. Uncovering such mechanisms would offer great expectations in the treatment of scars and therefore represents an important area of investigation. In this review, we provide a comprehensive summary of studies on injury‐induced skin regeneration within the foetus. The information contained in these studies provides an opportunity for new insights into the treatment of clinical scars based on the cellular and molecular processes involved.

## Graphical abstract

The schematic diagram shows the distinction between the healing process of the foetus and the adult mice after injury: the foetus of E16 shows less inflammatory cell infiltration, fibroblast colonization and ECM deposition than the adult mice. In addition, the participation of proteins and non‐coding RNAs (miRNAs, lncRNAs) also plays a key role in different prognosis.

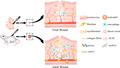

## 1 INTRODUCTION

Scars remain a challenging clinical problem. Adults often show multiple types of scars in response to trauma, burns, infections or surgery due to the fibrosis that can result from various reasons. The most troublesome pathological types are hypertrophic scars and keloids.^1^ A primary difference between these two is that hypertrophic scars are localized within the wound boundary, while keloids will grow beyond the edge of this boundary.^2^ Scars can result in both cosmetic and functional discomforts, such as itching or impaired mobility, but can also exert devastating psychologically effects.^2^, ^3^ Such psychological pressure often induces patients to seek effective means to beautify or reduce their scars. According to available reports, it has been estimated that up to tens of billions of dollars are expended for treatments and consequences of scars.^1^, ^4^

Despite the application of various methods for the treatment of hypertrophic scars or keloids, the effectiveness of these protocols remains quite limited. Accordingly, a better understanding of the mechanisms involved with scar formation is sorely needed. One potential approach to investigate the root cause of scars involves an examination of this process in the foetus. In the human foetus, a scarless wound repair process is present, a phenomenon that was reported over 30 years ago.^5^ Subsequent studies have revealed that this scarless wound repair depends on age and that there is a transition from a scar‐free to scar formation process that occurs in the later period of foetal development. This transition occurs at around 24 weeks in human pregnancy and at day 18 of gestation in mice.^6^, ^7^ This extraordinary ability for the initial prevention of scar formation followed by a transition to a scar formation process in the foetus suggests that adult skin may also possess a similar mechanism, which could be activated to reduce adult scar formation like that observed in the foetus.

**FIGURE 1:**
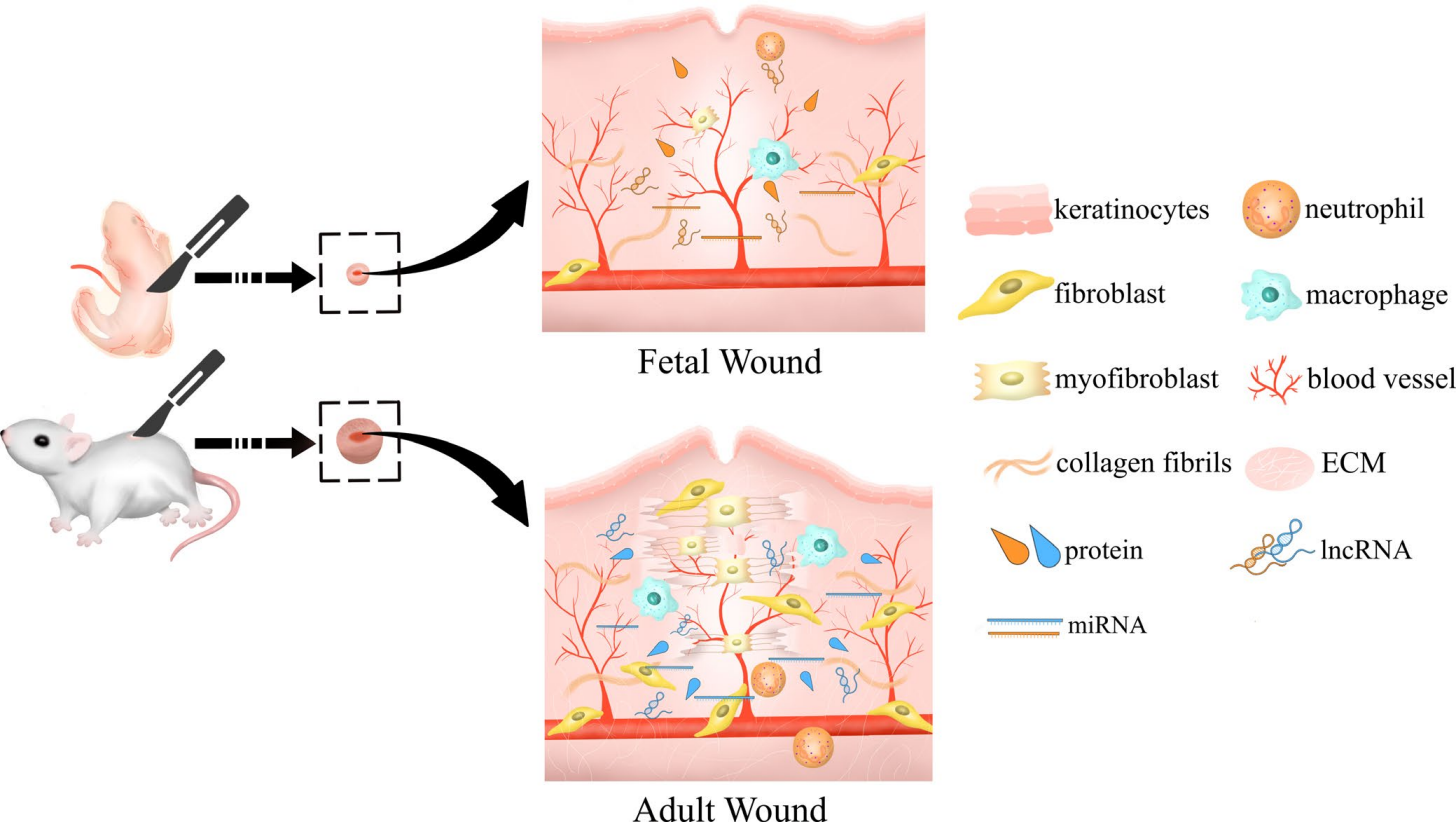
Differences and components involved in the repair process of foetal mice and adult mice after wound modelling

The molecular basis of scarless wound healing in mammalian foetuses has been partially reported before,^8^ but research on the basis of small rodent models has not been comprehensively sorted out. In this review, we summarized research mainly in the recent years on the healing mechanisms as observed in the foetus and adult from the perspective of tissues, cells and molecules, and explored the possibility of applying these findings to scar treatment. We focused on the wound healing process within the foetal model due to its remarkable capacity for scarless healing and evaluated the advantages and disadvantages that can be garnered from this model. Compared with the previous review,^9^ up‐to‐date findings on foetal skin and adult scars using gene sequencing technology, and mechanisms of various non‐coding RNAs in the formation of scars and their potential application for clinical treatment are also included in this review. Differences in tissue structure, cell types and molecules between foetal wound and adult wound are briefly illustrated in Figure 1.

## 2 EVOLUTION OF THE FOETAL WOUND MODEL

Since the initial discovery of foetal skin regeneration, a variety of animal wound healing models have been developed, including lamb, monkey, rabbit and opossum, with the goal that these models will provide an understanding of the mechanisms involved in this regeneration process.^10^, ^11^, ^12^ As one example, Stelnicki and colleagues used foetal lambs to evaluate the neural dependence of scarless healing after denervation.^13^ Although these large animal models are technically more advantageous, the expenses involved severely limit their use in these experiments. The substantially lower costs and shorter gestations of rats and mice, along with well‐identified genomes and transgenic technology that can be used to examine loss or gain of function in mice account for the preferential use of these rodent models.

A considerable amount of valuable information has been obtained from in vitro experiments using major functional cell lines of keratinocytes and fibroblasts subjected to injury.^14^, ^15^, ^16^ However, a major deficit in these studies was their failure to include all components of the skin and lack of assessing these injury responses in an in vivo environment.^17^, ^18^ Therefore, the development of an effective in vivo wound healing model is required for exploring the complex mechanisms involved with these responses.

**FIGURE 2:**
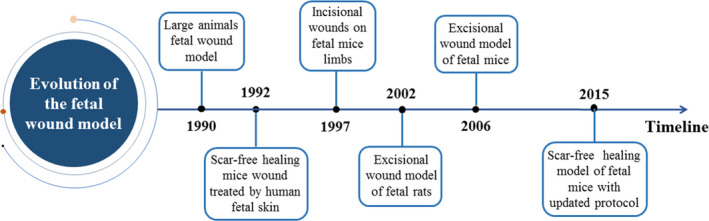
A chronological timeline showing key studies and models reported since the 1990s

In 1992, a graft of human foetal skin was observed to demonstrate scar‐free wound healing when applied onto the skin of mice. In this study, the amniotic fluid environment was not a necessary condition for this scar‐free regeneration of foetal skin.^19^ These findings provided the feasibility and foundation for the development of in vivo models to study foetal injury repair in mammals. One of the first attempts was that of Stelnicki and colleagues in 1997 and involved use of incisional wounds on the hind limbs of 14‐day‐old mice ^20^; however, the limited nature of their incision failed to affect the integrity of the skin in these mice, thus preventing an assessment of the healing process of this dermal deficiency. Over the next ten years, a number of in vivo models of excisional wounds were developed with a rat model in 2002 and the first in vivo excisional mouse model in 2006.^7^, ^21^, ^22^ The recent scar‐free wound healing model using foetal mice has been updated with the protocol of timed superovulation, while emphasizing the importance of amniotic fluid supplementation during surgery.^23^ In Figure 2, we show the exploration of foetal wound healing models, especially rat and mouse models, in the above‐mentioned time sequence since the 1990s.

## 3 COMPARISON BETWEEN FOETAL AND ADULT SKIN WOUND REPAIR

### 3.1 From macro to histological levels

Over the years, there have been a substantial number of studies directed at examining various aspects of tissue samples after foetal damage. In Table 1, we summarize the studies using the in vivo rat and mouse wound models that have been used to unearth the mechanisms of foetal skin regeneration.^22^, ^35^ Needless to say there are an extensive number of genes differentially expressed during foetal development, regardless of whether the foetus is damaged or not. With the maturation of gene sequencing technology, the mechanisms by which non‐coding RNAs participate in this process have also emerged. For example, dicer, the key enzyme for miRNA synthesis, is significantly increased in late embryonic tissue.^36^ A particularly interesting mouse model of relevance to this review is the African spiny mouse (Acomys), which shows regenerative responses in their skin following damage.^37^ These regenerative responses show a similar phenotype to that of the wound healing observed in the foetus with lower levels of inflammatory responses and no significant excessive amounts of collagen secretion.^38^, ^39^ When compared to normal mice, Acomys show differences in expression levels of phosphorylation‐related proteins, macrophage markers and immunomodulators after injury. Ubiquitination and phosphorylation are major mechanisms significantly enhanced in protein synthesis and degradation.^40^ Cellular and molecular differences after foetal injury will be discussed below.

**TABLE 1:** Summary of studies using the in vivo rat and mouse wound models

Scientists of study	Rat or mice; wound model	Time points of evaluation	Differentially expressed genes	Change trends of expression
Soo et al (2000)	Foetal rat; 2 mm full‐thickness excisional wounds	12, 36 and 72 h post‐wounding	Fibromodulin	Fibromodulin is up‐regulated in E16 rather than 19 wounds
Dang et al (2003)	Foetal rat; 2 mm full‐thickness excisional wounds	24, 48 and 72 h post‐wounding	MMP; TIMP	Greater MMP relative to TIMP expressed in E16 than E19
Traci et al (2004)	Foetal mice; 2 mm full‐thickness incisional wounds	6, 24 h and 7 d post‐wounding	COX‐2; PGE2	COX‐2 and PGE2 higher in E18 wound tissue than E15
Colwell et al (2006)	Foetal mice; 1 mm full‐thickness excisional wounds	8, 12 and 24 h post‐wounding	Lysyl oxidase	More lysyl oxidase in E19 compared with E17 wounds
Goldberg et al (2007)	Foetal mice; 3 mm full‐thickness excisional wounds	32 h post‐wounding	TGF‐β1; TβR‐2	TGF‐β1 and TβR‐2 increasing in rapid foetal wound closure
Goldberg et al (2007)	Foetal mice; 3 mm full‐thickness excisional wounds	32, 48 and 72 h post‐wounding	Procollagen types 1α2 and 3	Procollagen types 1α2 and 3 increased in E15 compared with the E18
Traci et al (2008)	Foetal mice; 2 mm full‐thickness incisional wounds	7 d post‐wounding	VEGF	Higher levels of VEGF in E18 than that in E15
Colwell et al (2008)	Foetal mice; 1 mm full‐thickness excisional wounds	1, 12 and 24 h post‐wounding	Genes detected by genomic microarray	Most of genes up‐regulated at 1 or 12 hours after injury in E17 compared with adult
Carter et al (2009)	Foetal mice; 2.5 mm full‐thickness excisional wounds	15 and 45 min post‐wounding	Cleaved caspase 7; cleaved PARP	Cleaved caspase 7 and cleaved PARP increasing more in E15 than in E18
Antony et al (2010)	Foetal rat; 2 mm full‐thickness excisional wounds	24 and 72 h post‐wounding	Genes detected by macroarray	Many neurodevelopmental genes up‐regulated in E16
Dardenne et al (2013)	Foetal mice; 2 mm full‐thickness incisional wounds	Ranging from 2 h to 7 d post‐wounding	Alarmin HMGB‐1	More HMGB‐1 expressed in E18 compared with E15
Zheng et al (2016)	Foetal rat; 2 mm full‐thickness excisional wounds	72 h post‐wounding	Fibromodulin; TGF‐β1	Fibromodulin reduces TGF‐β1 expression in E18 wounds
Hu et al (2018)	Foetal mice; 1 mm full‐thickness excisional wounds	1, 12 and 24 h	Pathway analysis of genes detected by microarray	Top 20 identified signalling pathways up‐regulated and down‐regulated at 1 and 12 h after injury; 11 up‐regulated pathways after 24 h
Wulff et al (2019)	Foetal mice; 2 mm full‐thickness incisional wounds	Ranging from 2 h to 7 d post‐wounding	Alarmin IL‐33	Higher level of IL‐33 expressed in E18 wounds compared with E15

### 3.2 Key roles of fibroblasts

Fibroblasts represent one of the most critical cells that participate in the entire process of wound repair through their effects on proliferation, differentiation, synthesis of collagen, interactions with other cells ^41^ and their capacity to form different fibrotic environments in various organs.^42^ Results obtained using sophisticated cell isolation methods ^43^ have revealed that enormous differences in gene expressions within fibroblasts are present between the mid‐ and late‐gestational periods in mice. These changes in fibroblasts at different developmental stages suggest that they may be closely related to the distinct responses observed after injury at these times,^44^, ^45^ as related to the accompanying activation and inhibition of multiple pathways.^46^ Cyclooxygenase 1 was one of the 12 differentially expressed inflammatory genes, which was expressed at higher levels in E18 versus E15 fibroblasts.^45^ When comparing human foetal versus adult dermal fibroblasts, as achieved with use of protein expression profiling, it was found that the heat‐shock cognate 71 kDA protein, which is active in organism survival, and tubulin alpha‐1A chain, a vital component of cell cytoskeleton, both showed greater expressions in foetal fibroblasts that conceivably account for the rapid regeneration of foetus, whereas cofilin‐1 and peroxiredoxin‐1 were expressed at comparatively higher concentrations in adult fibroblasts.^47^

An area of investigation that has attracted considerable attention of late is that of research on fibroblast heterogeneity and the tracking of different fibroblast lineages. It seems clear that distinct lineages can play diverse roles at different stages of skin development. For example, the reticular fibroblasts located in the lower portions of the dermis are responsible for synthesizing extracellular matrix and begin to repair immediately after injury. In contrast, papillary fibroblasts in the upper portion of the dermis regulate hair follicular formation and may be related to the loss of regional hair follicles after adult injury and play a role in the later stages of wound healing.^48^, ^49^ As based on the gene, engrailed 1 (En1), which is expressed in embryonic cells, two related fibroblast cell lines have been identified, En1‐lineage‐past fibroblasts (EPFs) and En1‐lineage‐naive fibroblasts (ENFs).^50^ Interestingly, these two cell lines exert markedly different effects upon scar formation, with EPFs primarily contributing to the formation of pathological scars, while ENFs are not involved in this process but are closely related to dermal development and regeneration. The number of ENFs gradually decreases with development, which is in line with the decline in regeneration effects and the increase in scar formation as observed in response to damage at later stages of embryonic development. More interestingly, the scarring effect of EPFs can be reversed by localized treatments of ENFs.^51^

During wound healing, myofibroblasts, derived from the differentiation of fibroblasts, play a role in the synthesis of ECM and tissue contraction.^52^ It has been reported that foetal skin wounds lack myofibroblasts ^8^; however, application of TGF‐β1 onto human foetal fibroblasts cultured in vitro induced cells, which obtained similar characteristics to myofibroblasts.^53^ Strangely, myofibroblasts, which have always been considered as fully differentiated cells, can aid in fat reconstruction.^54^ When single‐cell sequencing technology was utilized to detect the presence of 12 heterogeneous fibroblasts in the wounded skin of adult mice, a subcluster of fibroblasts derived from bone marrow was found. Results, as observed with bone marrow transplantation technology, have demonstrated that bone marrow haematopoietic cell lines can generate myofibroblasts and regenerate adipocytes, which may be related to the dilemma of fat regeneration after trauma.^55^

### 3.3 Differences in keratinocytes

As a critical component of the epidermis, keratinocytes secrete keratin filaments to participate in the keratinization and protection of skin from the invasion of external microorganisms.^56^ Of particular significance to the present review, keratinocytes also undertake the major mission of re‐epithelization and wound closure after skin damage ^57^ and are a key factor in the delay of wound healing in adults.^58^ Pastar and colleagues reported some notable changes that occur in keratinocyte genes during embryonic development with 24 genes being down‐regulated in keratinocytes at E16 as compared to E18 and no obvious evidence for any genes being up‐regulated. In E18 keratinocytes, specifically up‐regulated β‐catenin–dependent Wnt pathways were demonstrated to play a key role in the scarring process of late‐gestational age.^44^ In contrast, focal adhesion kinase (FAK) was previously manifested a close connection with mechanotransduction and fibrosis. Interestingly, keratinocyte FAK‐deleted mice were found to obtain thinner skin and lessened collagen density, while FAK‐deleted keratinocytes actively participated in mechanotransduction and extracellular matrix production, by overexpressing Igtbl, Mmpla and Col4a1.^59^, ^60^

Receptor‐interacting protein kinase 4 (RFPK4) ^61^, ^62^ and interferon regulatory factor 6 (IRF6) ^63^, ^64^, ^65^ both play a vital role in the differentiation of keratinocytes, with mutations in either of these two having the potential of resulting in epidermal developmental disorders.^66^, ^67^, ^68^ As compared to that observed in RIPK4 full knockout mice, in neonatal mice with a specific removal of RIPK4 in keratinocytes, the epidermis shows a normal structure, but the lack of a skin barrier function leads to accelerated water loss and severely limits the lifespan of these newborns.^69^, ^70^ IRF6 interacts with the AP2P protein and MCS9.7 enhancer to form a regulatory network, which together guides epidermal development and is a risk factor associated with orofacial clefts.^71^ IRF6 also appears to be involved with the capacity for epidermal progenitor cells to produce regeneration in portions of sweat glands after burns.^72^ In addition to their individual effects, these two factors interact with each other as RIPK4 can regulate IRF6 to alter the expression of downstream transcription factors, which forms an axis in the protein kinase C pathway to guide the differentiation of keratinocytes.^73^ This axis, in turn, boosts inflammation within the epidermis by inducing an excessive production of pro‐inflammatory keratinocyte cytokines, such as CCL5 and CXCL11.^74^ With the use of RNA‐seq, ChIP‐seq and ATAC‐seq, Oberbeck and colleagues have demonstrated the gene enrichment present in IRF6 knockout foetal mice during development, along with the general and histological differences in these mice as compared to normal controls.^75^ As compared to that observed in adults, human foetal keratinocytes expand faster, show properties similar to that of stem cells and were induced to demonstrate an orderly differentiation as demonstrated in vitro. These findings suggest that human foetal keratinocytes can be used as bioengineering material for cell transplantation to assist in wound healing.^76^, ^77^, ^78^

### 3.4 Other cells emerging to be targeted

In addition to fibroblasts and keratinocytes, there exist a number of other molecules and cells that show contrasting temporospatial characteristics and expressions in wound responses between the adult and foetus. For example, observations from immunofluorescence have revealed that human foetal skin contains significantly reduced amounts of immune cells and chemokines, such as macrophages, mast cells, CCL17 and CCL21, as compared to that observed in adult skin. The number of leucocytes marked by CD45 is also decreased in skin samples from the foetus.^79^ The weak degranulation of immature mast cells that promotes scar‐free wound healing in foetal mice can be reversed with the addition of exogenous mast cell lysate.^80^ Mast cells play a significant role in the scar formation process, and their activation promotes excessive collagen secretion and abnormal deposition,^81^ while blocking mast cell function inhibits scarring without delaying the wound healing process.^82^

In addition to these components of foetal skin that can modulate this wound healing process, the amniotic fluid, cord blood and amniotic membrane present during pregnancy contain a rich source of stem cells, which can also contribute to the repair and scar‐suppressing effects.^83^, ^84^ Both mesenchymal stem cells, derived from human umbilical cord blood (UCB‐MSCs), and stem cells, derived from human amniotic fluid (hAFS), enable adult injuries to acquire foetal‐like repair processes,^85^, ^86^ which were respectively characterized by reduced level of inflammatory cytokine expressed by UCB‐MSCs and closely analogous collagen type III proportion to that of the foetal wound triggered by hAFs. Simultaneously, exosomes, as part of the paracrine activity of stem cells, also promote wound healing and limit scar formation.^87^, ^88^ Recent evidence indicates that peripheral cells play a role in scar formation and regulation of vascular regeneration.^89^ As peripheral cell therapy has been shown to reduce the accumulation of inflammatory cells and organize collagen in remodelling phases, this suggests one possible mechanism for this effect of peripheral cells in this scar formation.^90^

## 4 INVOLVEMENT OF DIVERSE MOLECULES

### 4.1 Proteins displaying key functions

In foetal sheep, a diverse array of molecular responses has been observed in response to wounds and trauma. For example, IL‐10 effectively reduces the number of inflammatory cells and significantly lessened scars at the location of a severe trauma.^91^ IL‐4Rα–activated macrophages participate in the fibrosis process and up‐regulate the expression and deposition of collagen fibres through the continuous secretion of lysine hydroxylase 2 in fibroblasts.^92^ The plasminogen activator inhibitor type 1 (PAI1), secreted by keratinocytes, stimulates intercellular crosstalk between fibroblasts and mast cells in adhesions, which likely contributes to scleroderma.^93^ As demonstrated both in vivo and in vitro, inhibition of calpain constrains granulation tissue growth by reducing collagen synthesis.^94^ Considering that inhibitors of focal adhesion kinase (FAK) can suppress scar formation, Kun et al developed a controlled drug release of FAK, which provides an innovative approach for clinical treatment.^95^ Fibromodulin, a small molecule that can effectively inhibit scar formation, is activated in both embryonic and adult stages through the TGF‐β pathway.^33^, ^96^ In fibromodulin knockout mice, the combination of an up‐regulation of the type I TGF‐β receptor during the inflammation response phase and increases in TGF‐β3 and TGF‐β receptors during the proliferation phase extends wound healing time and maintains a source for scarring.^97^ FOXO1 knockout or local interference in its expression accelerates wound healing and reduces fibrosis. Moreover, FOXO1 is highly concentrated within the borders of lesions in keloid patients, suggesting an invasion into normal skin.^98^

### 4.2 Non‐coding RNAs implicated in dermal fibrosis

Along with this research focusing on proteins, in recent years, the emergence of non‐coding RNAs, which actively participate in every stage of wound healing, has also attracted a considerable amount of attention.^9^ Gene therapy involved with the application of non‐coding RNAs will inevitably represent a major breakthrough in the clinical treatment of various diseases. With regard to skin fibrosis, the mechanisms responsible for the extreme hyperplastic scar and keloid in trauma sequelae are closely related to non‐coding RNAs, in particular miRNA and lncRNA. Accordingly, the mechanisms of non‐coding RNA involvement in scar development and their prospects for being targeted in clinical treatment warrant discussion.

#### 4.2.1 Effects of miRNAs on scar formation

miRNAs are small molecules of approximately 22‐nucleotide lengths, which target the inhibition of mRNA translation. Interestingly, miRNAs are characterized by dynamic changes during embryonic development and usually suppress expression of factors in foetal wounds.^99^ In Table 2, we present a summary of research primarily focused on the impact of miRNAs in scar formation during the adult stage.^100^, ^101^, ^102^, ^103^, ^104^, ^105^, ^106^, ^107^, ^108^, ^109^, ^110^, ^111^, ^112^, ^113^, ^114^, ^115^, ^116^, ^117^, ^118^, ^119^, ^120^ Differentially expressed miRNAs have been detected between scar lesions in adult versus embryonic skin.^36^, ^121^ Such discriminatory responses in core miRNAs as related to scar development after injury suggest promising new targets for clinical treatment.

**TABLE 2:** Summary of miRNAs actively involved in scar growth after injury

miRNA	Scientists of study	Correlation with scar growth	Targets involved	Effect
miR‐29b	Guo et al (2017)	Negative	TGF‐β1, Smad, CTGF	In vivo: reduces inflammation, collagen synthesis and deposition
miR‐495	Guo et al (2019)	Negative	FAK	In vitro: reduces fibroblast proliferation and enhances apoptosis In vivo: reduces collagen synthesis
miR‐98	Bi et al (2017)	Negative	Col1A1	In vitro: reduces fibroblasts viability and enhances apoptosis
miR‐149	Lang et al (2017)	Negative	IL‐1α, IL‐1β, IL‐6, TGF‐β3, Col3	In vitro: reduces inflammatory factor expression in HaCaTs In vivo: reduces inflammation and collagen deposition
miR‐143‐3p	Mu et al (2016)	Negative	CTGF	In vitro: reduces ECM synthesis including Col1, Col3 and α‐SMA and reduces fibroblasts proliferation in the Akt/mTOR pathway
MicroRNA Seq‐915_x4024	Zhao et al (2019)	Negative	Sar1A, Smad2, TNF‐α, IL‐8	In vitro: enhances keratinocyte proliferation and migration and reduces inflammation In vivo: reduces inflammatory cell infiltration and scar size
miR‐519d	Zhou et al (2018)	Negative	SIRT7	In vitro: reduces fibroblast proliferation and ECM synthesis including Col1, Col3 and α‐SMA and enhances apoptosis
miR‐145‐5p	Shen et al (2020)	Negative	Smad2, Smad3	In vitro: reduces fibroblast proliferation and migration In vivo: reduces inflammation and fibrosis
miR‐145	Zhu et al (2015)	Negative	Smad3	In vitro: gene up‐regulated by PPAR‐gamma agonist reduces collagen synthesis
miR‐145	Gras et al (2015)	Positive	Col1A1, TGF‐β1	In vitro: regulates differentiation from fibroblast to myofibroblasts, enhances myofibroblast viability and migration.
miR‐6836‐3p	Liu et al (2018)	Positive	CTGF	In vitro: enhances fibroblast proliferation and reduces apoptosis
miR‐21	Guo et al (2017)	Positive	COl1A1, COl1A2, fibronectin	In vitro: enhances fibroblast proliferation and reduces apoptosis In vivo: antagomir reduces collagen deposition and scar size
miR‐181b	Kwan et al (2015)	Positive	Decorin	In vitro: regulates differentiation from fibroblast to myofibroblasts
miR‐192	Li et al (2017)	Positive	SIP1	In vitro: enhances ECM synthesis including Col1, Col3 and α‐SMA In vivo: enhances collagen synthesis
miR‐181b‐5p	Liu et al (2019)	Positive	Decorin	In vitro: enhances fibroblast proliferation and reduces apoptosis in the MEK/ERK/p21 pathway
miR‐152‐3p	Wang et al (2019)	Positive	FOXF1	In vitro: enhances fibroblast proliferation and ECM synthesis including Col1, Col3 and fibronectin
miRNA‐1908	Xie et al (2016)	Positive	Scar suppressor Ski	In vitro: enhances fibroblast proliferation and pro‐inflammation In vivo: increases scar size
miR‐155	Velazquez et al (2017)	Positive	IL‐1β, TNF‐α, IL‐10, α‐SMA, Col1, Col3	In vivo: gene knockout reduces inflammation and ECM synthesis including Col1, Col3 and α‐SMA without healing time compensation
miR‐130a	Zhang et al (2019)	Positive	CYLD	In vitro: enhances fibroblast proliferation and ECM synthesis including Col1, Col3 and α‐SMA in the Akt pathway In vivo: gene inhibitor reduces collagen deposition
miR‐203	Zhou et al (2018)	Positive	Hes1	In vitro: promotes differentiation from ESCs to myofibroblasts In vivo: accelerates healing and reduces scar size
miRNA‐21, miRNA‐141‐3p, miRNA‐181a, miRNA‐205	Lyu et al (2019)	Positive	–	In vitro: enhances fibroblast proliferation and reduces apoptosis in the PI3K/Akt pathway
miRNA‐637, miRNA‐1224	Lyu et al (2019)	Negative	–	In vitro: enhances fibroblast proliferation and reduces apoptosis in the TGF‐β1/Smad3 pathway

#### 4.2.2 Complex mechanisms of lncRNAs

The mechanisms of long non‐coding RNAs are more intriguing, but complicated, and have recently been widely investigated in the pathogenesis of skin fibrosis, especially in hypertrophic scars and keloids. Based on results obtained with modern gene sequencing or chips, a number of lncRNAs have been identified, which could serve as biomarkers in the course of various diseases; however, their mechanisms remain unclear.^122^, ^123^ Moreover, through in vitro loss‐of‐function or gain‐of‐function experiments, it is possible to distinguish lncRNAs that promote or inhibit the development of hypertrophic scars or keloids.

The sponging effect of lncRNAs on miRNAs forms an axis that determines the downstream mRNA expression. These axes including lncRNA‐ATB/miR200c/TGF‐β2, lncRNA XISt/miR‐29b‐3p, lncRNA HOXA11‐AS/miR124‐3p/TGF‐βR1 and lncRNA‐H19/miR‐29a/Col1A1 have been shown to promote pathogenesis in vitro, through enhancing fibroblasts of vitality, proliferation and migration or by ECM synthesis while inhibiting apoptosis.^124^, ^125^, ^126^, ^127^, ^128^ In contrast, the axes lncRNA COl1A2‐AS1/miR‐21/smad7, lncRNA 8975‐1 and lncRNA AC067945.2 inhibit scar formation through exerting opposite effects on fibroblast pathological changes and collagen synthesis as that observed in scar formation.^128^, ^129^, ^130^ Moreover, in cell co‐culture experiments, lncRNA ASLNCS5088 exosomes as secreted from M2 cells endogenously bind miR‐200c‐3p, thus promoting fibroblast differentiation and pathological function, an effect which can be reversed by GW4869.^131^ As these complicated functions of lncRNAs are gradually revealed, their application and effectiveness in the treatment of hypertrophic scars and keloids will be realized.

## 5 CONCLUSIONS

Research on scarless wound healing and scar reduction has been ongoing for decades, with innovative techniques and in vitro and in vivo models used to identify numerous cells, factors and novel genes related to this process. Armed with this information, as well as findings from future studies, effective clinical treatment, such as targeted drugs for key molecules or the development of engineered cells, will gradually be revealed. However, the complex and overlapping pathways may require more investigations on the regulation mechanisms of genes with unidentified functions. Newly emerging technologies focusing on detection of non‐coding RNA, single‐cell transcriptome and RNA methylation can be regarded as a breakthrough in further exploration of unknown areas. Even if the impeccable regeneration of foetal skin cannot be fully exerted in clinical practice, at present, we feel this review will promote the evolution of this process to bring new perspectives for future treatment and cosmetology.

## CONFLICTS OF INTERESTS

The authors have no conflicts of interest to declare.

## AUTHOR CONTRIBUTIONS

Jia‐Li Yin involved in writing—original draft preparation. Jia‐Li Yin, Yan Wu, Zheng‐Wei Yuan and Xing‐Hua Gao involved in writing—review and editing. Zheng‐Wei Yuan and Xing‐Hua Gao involved in visualization. Xing‐Hua Gao and Hong‐duo Chen involved in supervision, project administration and funding acquisition.

## Data Availability

The data that support the findings of this study are available from the corresponding author upon reasonable request.
